# CASE REPORT Treatment of a Lower Extremity Lymphocele With Intraoperative Lymphatic Mapping

**Published:** 2013-11-07

**Authors:** Caleb P. Canders, Phuong D. Nguyen, Jaco H. Festekjian, George H. Rudkin

**Affiliations:** ^a^Divisions of Emergency Medicine, UCLA Medical Center, Los Angeles, Calif; ^b^Plastic and Reconstructive Surgery, UCLA Medical Center, Los Angeles, Calif

**Keywords:** isosulfan blue dye, lower extremity, lymphatic mapping, lymphocele, resection

## Abstract

**Objective**: Lower extremity lymphoceles secondary to saphenous vein grafting are exceptionally rare and there is only 1 previously reported case in the English literature. Data on treatment of lower extremity lymphoceles are limited and based on studies of groin lymphoceles. We discuss operative resection with selective ligation of feeding lymphatic vessels as a treatment option of lower extremity lymphoceles. **Methods**: A 64-year-old man who had undergone coronary artery bypass grafting 6 years prior presented with a left lower extremity mass at the site where his saphenous vein had been harvested. Examination demonstrated a 12-cm, mobile, nonpulsatile mass at his medial left calf. The findings of magnetic resonance imaging were consistent with a lymphocele. **Results**: Intraoperative injection of isosulfan blue dye was used to identify feeding lymphatic vessels and the lymphocele cavity was excised. Leg drains were discontinued after 3 days, and the patient was discharged home after 6 days. **Conclusion**: Operative resection with isosulfan blue dye lymphatic mapping and selective ligation of lymphatic vessels is a viable treatment of lower extremity lymphoceles.

Groin lymphoceles resulting from intraoperative disruption of lymphatic vessels are a common complication in patients undergoing femoral arterial operations. In contrast, only 1 case of a lower extremity lymphocele following such a procedure has been reported in the English literature.^1^ Lymphoceles in general are associated with increased wound complications and longer hospital stays.^2^^,^^3^ Resection with intraoperative lymphatic mapping using isosulfan blue dye (Lymphazurin 1%, Ben Venue Labs, Inc, Bedford, Ohio) has been shown to be an effective treatment for groin lymphoceles; however, there are virtually no data on treatment options for lower extremity lymphoceles.^4^

We describe a patient who developed a lower extremity lymphocele following saphenous vein harvesting. The lymphocele was resected and intraoperative injection of isosulfan blue dye was used to identify and selectively ligate the feeding lymphatic channels. We demonstrate that this technique, which has previously been described in the treatment of groin lymphoceles, can be adapted to treat lower extremity lymphoceles.

## METHODS

**Figure 1:**
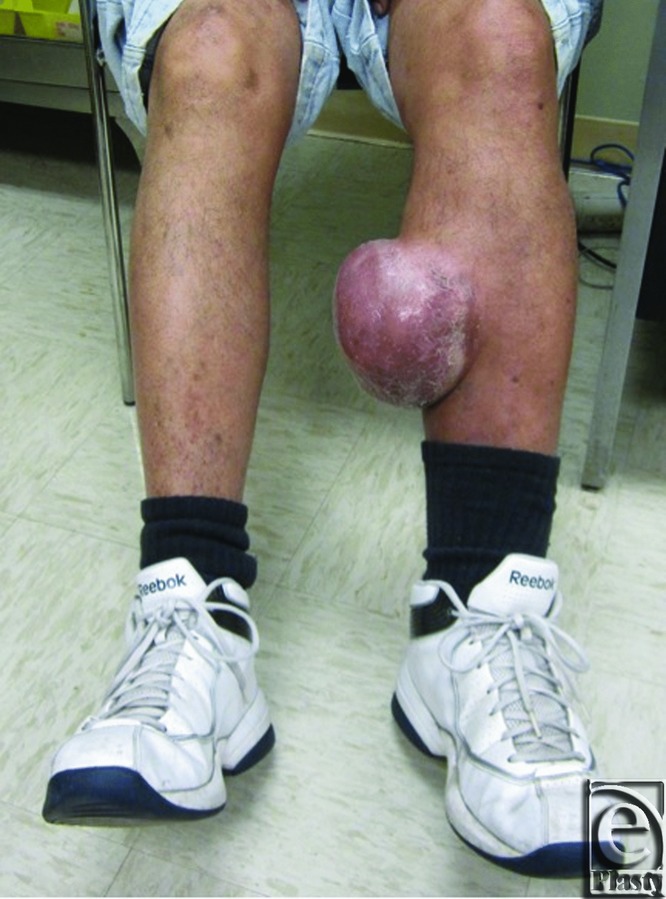
Left medial calf lymphocele at site of harvested saphenous vein.

**Figure 2:**
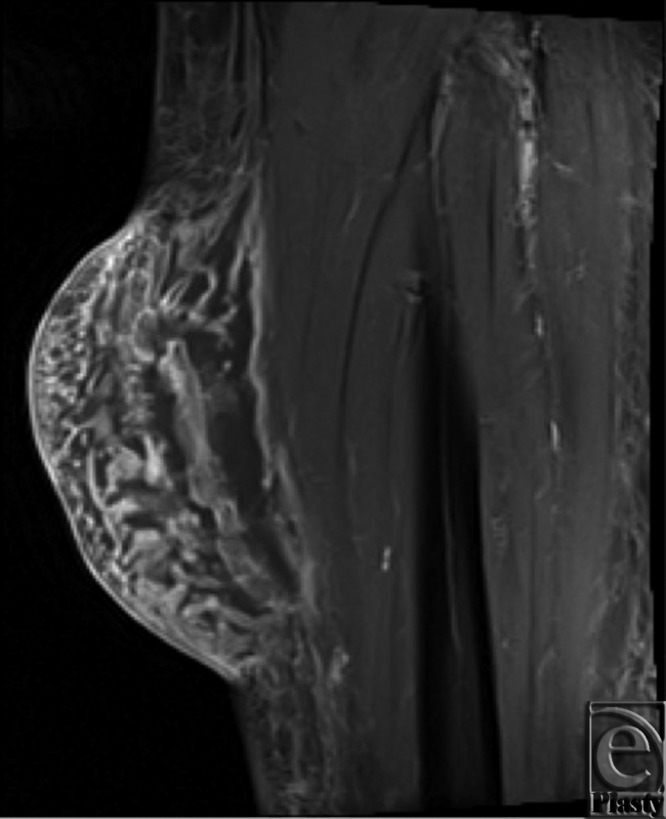
Subcutaneous soft tissue mass measuring 12.0 × 10.3 × 4.7 cm^3^ in the vicinity of prior greater saphenous vein, without abnormal enhancement.

A 64-year-old man who had undergone coronary artery bypass grafting 6 years prior presented with a left lower extremity mass at the site where his saphenous vein had been harvested. The mass developed approximately 6 months postoperatively, had progressively increased in size, and was associated with dull discomfort. The patient denied local trauma, discharge, swelling elsewhere in his extremities, fevers, or chills. His examination was remarkable for a 12-cm, mobile, nonpulsatile soft tissue mass at his medial left calf without tenderness or erythema (Fig 1). Lower extremity ultrasonography demonstrated focal subcutaneous edema, and magnetic resonance imaging showed a 12.0 × 10.3 × 4.7 cm^3^ subcutaneous soft tissue mass with surrounding fibrous tissue in the vicinity of his prior greater saphenous vein (Fig 2). The patient was diagnosed with a chronic lower extremity lymphocele and was taken to the operating room for resection and drain placement.

**Figure 3:**
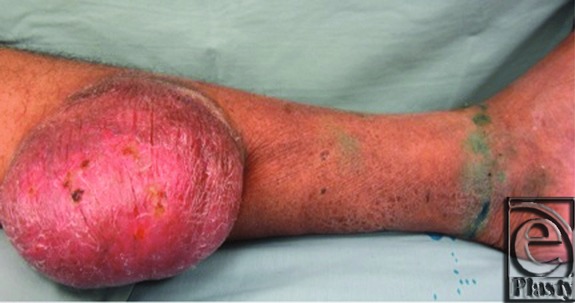
Intradermal injection of isosulfan blue dye circumferentially at the ankle.

**Figure 4:**
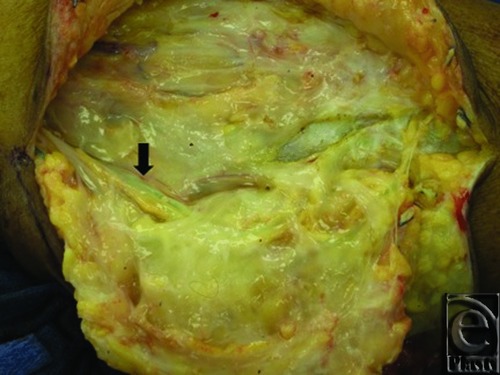
A blue-staining lymphatic vessel (arrow) is identified within the exposed lymphocele cavity.

The operative technique was comprised of preoperative intradermal injection of 4 mL of 1% isosulfan blue circumferentially around the ankle (Fig 3). The leg was then elevated for 10 minutes. The lymphocele was incised and the full extent of the cavity was exposed. Several blue feeding lymphatic channels were identified and isolated (Fig 4). These lymphatics were ligated with either braided absorbable sutures or vascular metallic clips. The lymphocele was then dissected and excised circumferentially off the gastrocnemius fascia. Two wound drains were placed through separate proximal incisions. The wound was irrigated and closed in multiple layers.

## RESULTS

Postoperatively, the wound was covered with gauze dressing and wrapped in a circumferential elastic bandage from the toes to the knee. The patient was kept on bedrest with his leg elevated above the level of the pelvis for the first 3 postoperative days. Activity was liberalized in a graduated fashion thereafter. The leg drains were discontinued on postoperative day 5 and the patient was discharged home on hospital day 6. He continued to wrap the leg with elastic bandage for 4 weeks. The patient developed no wound complications, allergic reactions to the isosulfan blue dye, or recurrence of his lymphocele.

## DISCUSSION

Lymphoceles occur when afferent lymph vessels are disrupted and lymph fluid accumulates in a potential space without a distinct epithelial lining.^5^ Lymphatic complications after femoral arterial reconstructive operations are common and groin lymphoceles are estimated to develop in 1.5% to 8% patients, depending on surgical technique.^6^^,^^7^ However, the development of a lower extremity lymphocele after saphenous vein grafting is extremely rare and only 1 case has been previously reported in the English literature.^1^ Lymphatic complications in general are associated with an increased length of hospital stay of up to 30 days, wound infection in 57% patients, and increased wound dehiscence.^2^^,^^3^

There is limited data on diagnosis and treatment of lower extremity lymphoceles. Lymphoscintigraphy is useful in the diagnosis of groin lymphoceles, however, no data exist on its use in diagnosis of lower extremity lymphoceles and it is a highly invasive, high-cost, and low-resolution study.^8^ In cases of groin lymphoceles, treatment options include observation, serial aspiration and compression, instillation of sclerosing agents, radiation therapy, negative pressure wound therapy, and operative resection of the cavity with or without muscle flap coverage.^3^^,^^9^^-^19 Although there is no standardized management, there is evidence that early wound exploration with selective lymphatic ligation leads to shorter hospitalizations and fewer wound complications than conservative therapy.^3^^-^4^,^^15^ Microsurgical construction of lymphatic-venous anastomoses is an alternative surgical treatment of groin lymphoceles, however, patients may experience postoperative lymphedema and this technique has not been used in lower extremity lymphoceles.^8^

Isosulfan blue dye binds albumin in the interstitial fluid and is taken up selectively by the lymphatic system. It has been used since the 1970s to treat lymphatic complications, however, this is its first reported use in the management of a lower extremity lymphocele. In patients with all types of lymphatic complications, isosulfan blue dye identifies approximately 90% disrupted lymphatic vessels.^15^^,^^20^ In a case series of patients with groin lymphoceles, the use of isosulfan blue dye facilitated identification and ligation of 100% disrupted lymphatic vessels and no patients had a recurrence of their lymphocele.^4^ Intraoperative injection of isosulfan blue dye in patients with groin lymphoceles has also been shown to decrease mean hospital stay by 4 to 17 days, decrease total duration of therapy by 28 to 56 days, and decrease wound infections by more than 50% compared to nonoperative therapy alone.^3^^,^^20^ Isosulfan blue dye is relatively safe and the majority of adverse events are skin reactions, including permanent skin pigmentation. However, it should be noted that potentially life-threatening hypotension occurs in 0.75% patients.^21^

## CONCLUSION

Lower extremity lymphoceles are rare but should be suspected in patients with the appropriate surgical history and physical examination. This case demonstrates that operative resection with intraoperative isosulfan blue dye lymphatic mapping and selective ligation of lymphatic vessels is a viable treatment option of lower extremity lymphoceles.
